# Supplementary figures and images for: Qualitative Differences Between the IFNα subtypes and IFNβ Influence Chronic Mucosal HIV-1 Pathogenesis
Source: PLoS Pathog. 2020 Oct 16;16(10):e1008986. doi: 10.1371/journal.ppat.1008986 (PMC7592919; doi:10.1371/journal.ppat.1008986)

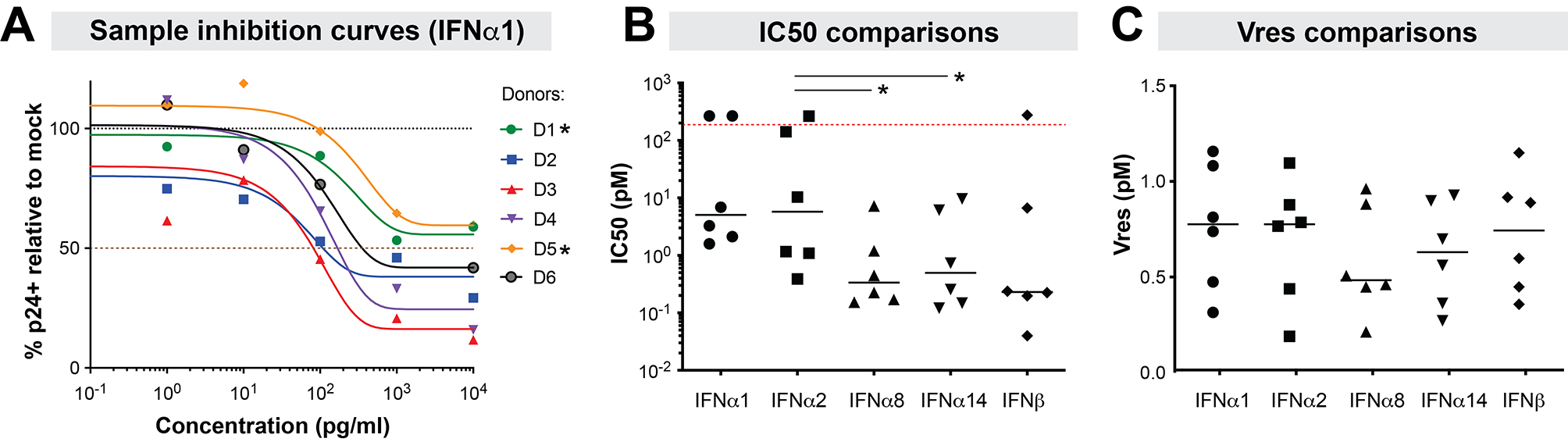

Supplement: S1 Fig — (A) HIV-1 inhibition curves for 6 different donors with IFNα1 based on a one-phase decay equation. Note that 2 donors (asterisk) did not reach 50% inhibition (above the red dashed line). The IC50s for these samples were calculated as 10,000 pg/ml, the highest dose used. (B) IC50s were calculated based on the sigmoidal plot and converted to pM. Each dot corresponds to a different LPMC donor. A few datapoints did not reach an IC50. The difference in IC50 between IFNα2 and either IFNα8 or IFNα14 were significant (*p<0.05) based on 2-tailed Wilcoxon matched pairs test (GraphPad Prism 5.0). (C) The residual virus replication at maximum IFN-I doses (Vres) was calculated for each donor based on the plateau of the best-fit equation. The differences were not significant based on a one-way ANOVA using Friedman’s test (p>0.05). For panels (B) and (C), the central line corresponds to the median values. (JPG) [file ppat.1008986.s001.jpg]

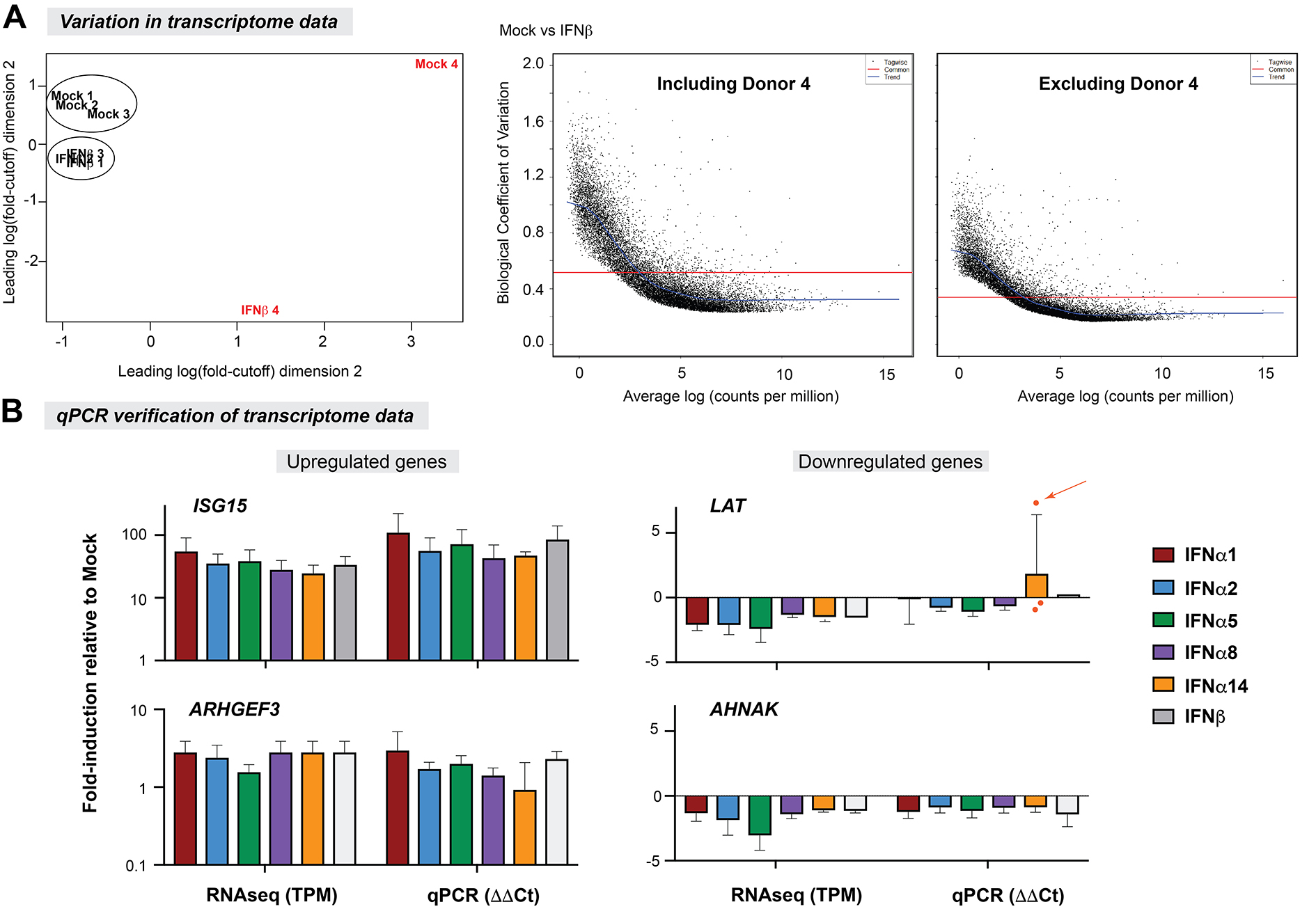

Supplement: S2 Fig — (A) (Left panel) Multidimensional scaling plot of distances between gene expression profiles of different LPMC donors, using mock and IFNβ-treated data as an example. Donor 4 is highlighted in red. (Right panels) Inclusion of donor 4 increased the biological coefficient of variation (BCV) when comparing mock to IFN-I treatment. As an example, BCV plots with or without donor 4 are shown for mock vs IFNβ treatment. The red line corresponds to the common dispersion for all genes; the blue curve is the dispersion trend; each black dot is the genewise dispersion rate. Higher dispersion trends for mock versus IFNα1, IFNα2, IFNα5, IFNα8 and IFNα14 were also observed if donor 4 was included (not shown). These were used to justify exclusion of donor 4 from subsequent analysis. (B) Confirmation of transcriptome data via qPCR. Fold-induction relative to mock values were compared for 4 genes between the RNASeq data (based on TPM values) and qPCR (based on ΔΔCt method). Note that one outlier in the LAT qPCR for IFNα14 (orange arrow) drove a positive fold induction with a large error bar. The individual data points are 7.03, -1.00 and -0.44. (JPG) [file ppat.1008986.s002.jpg]

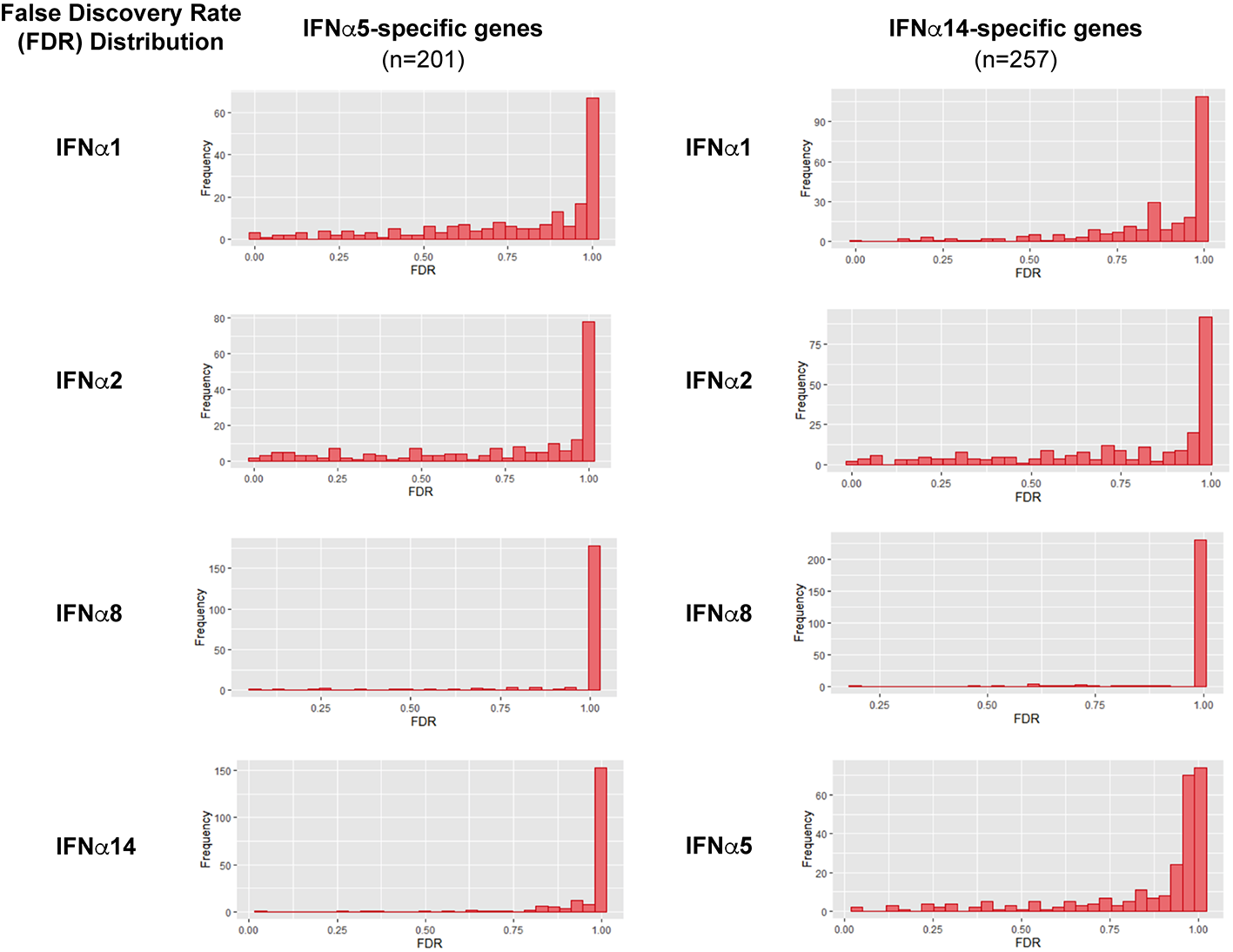

Supplement: S3 Fig — (Left panels) IFNα5-specific genes (n = 201) were classified based on significant induction/suppression in IFNα5-treated cells relative to mock at a 20% FDR cut-off. However, when these genes were evaluated in transcriptome datasets for IFNα1, IFNα2, IFNα8 and IFNα14, most had FDR values >90%, suggesting that these IFNα5-specific genes were not even close to being statistically-significant in these other IFNα subtypes. (Right panels) Evaluation of IFNα14-specific genes against gene datasets for IFNα1, IFNα2, IFNα8 and IFNα5. Bars correspond to the frequency of genes that fell within the FDR values noted in the x-axis. (TIF) [file ppat.1008986.s003.tif]

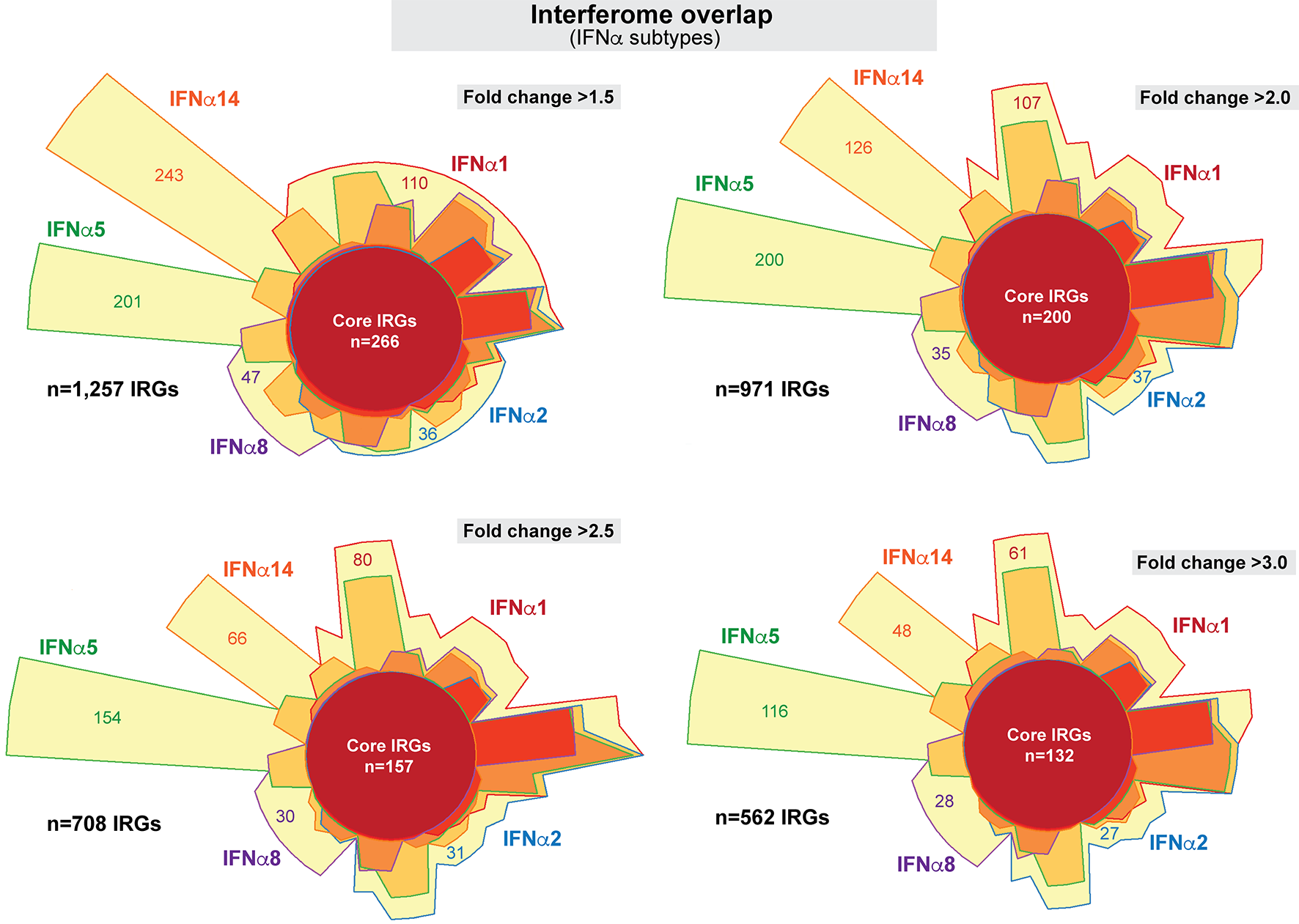

Supplement: S4 Fig — IFN regulated genes (IRGs) in primary gut CD4+ T cells (n = 3 donors) were determined via RNAseq. Euler diagrams are used to show the overlap between IFNα subtype interferomes at 1.5, 2.0, 2.5 and 3.0 fold-change (FC) cut-offs. The number of IFN-regulated genes (IRGs) decrease with higher FC cut-offs, but genes unique to each IFNα subtype remained significant. (TIF) [file ppat.1008986.s004.tif]

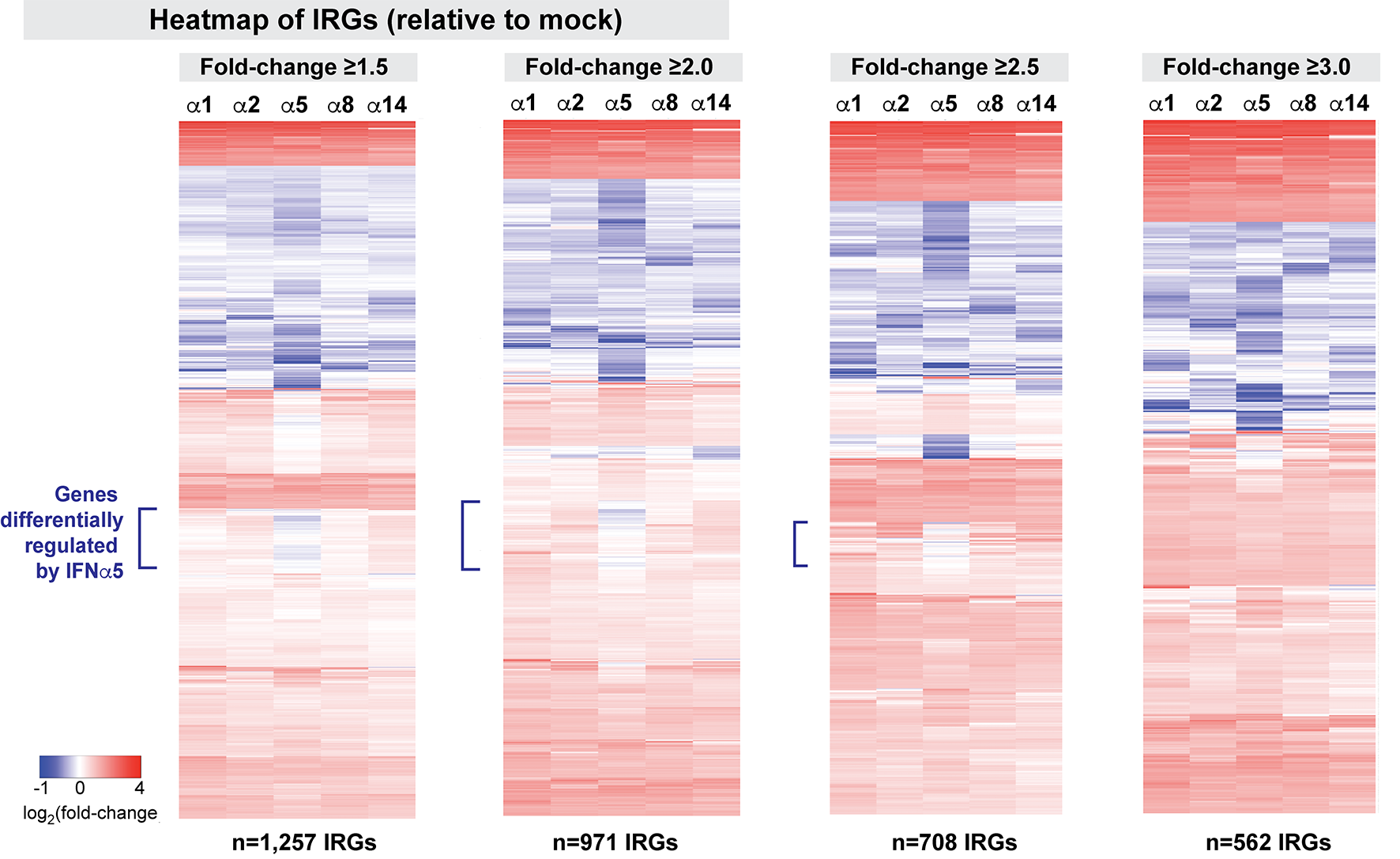

Supplement: S5 Fig — Heat maps of genes that were differentially regulated by various IFN-Is in primary gut CD4+ T cells (n = 3 donors) at various fold-change cut-offs. Red bars correspond to upregulated genes relative to mock, whereas blue bars correspond to genes that were downregulated. Brackets indicate genes that were weakly downregulated by IFNα5, but upregulated by IFNα1, 2, 8 and 14. These IFNα5-differentially regulated genes were absent at a fold-change cut-off of 3.0. (TIF) [file ppat.1008986.s005.tif]

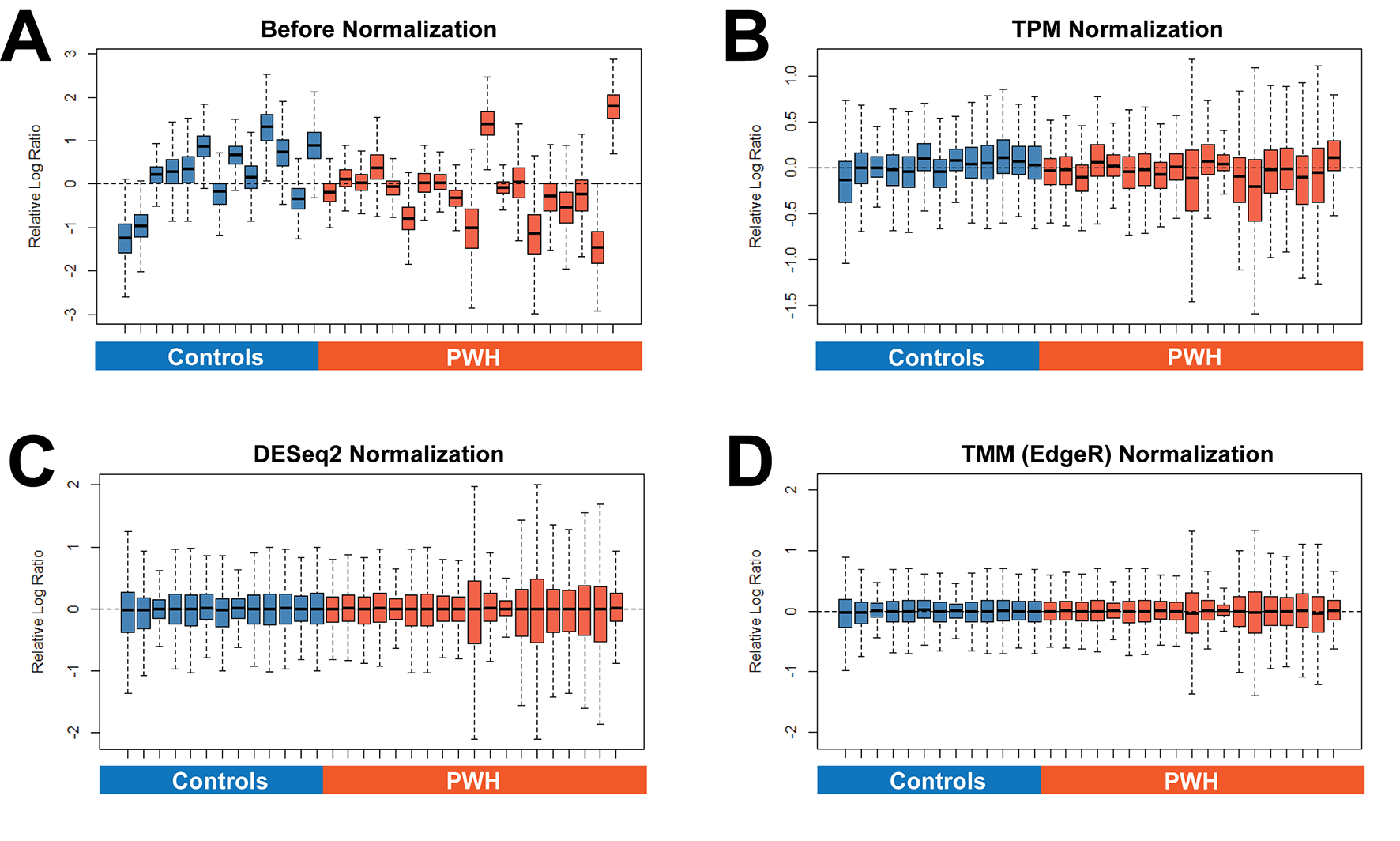

Supplement: S6 Fig — Visualization of unwanted variation of filtered RNAseq data in clinical biopsy samples from HIV uninfected (n = 13) and PWH (n = 19) samples using Relative Log Expression (RLE) Plots. (A) Without normalization; (B) Normalization via Transcripts per Million; (C) DESeq2; and (D) Trimmed Mean of M-values (TMM) using edgeR. The RLE plot, which is a boxplot of deviations from gene medians, shows the TMM normalization is the best in our case, based on the position at 0 of medians and narrow widths. (TIF) [file ppat.1008986.s006.tif]

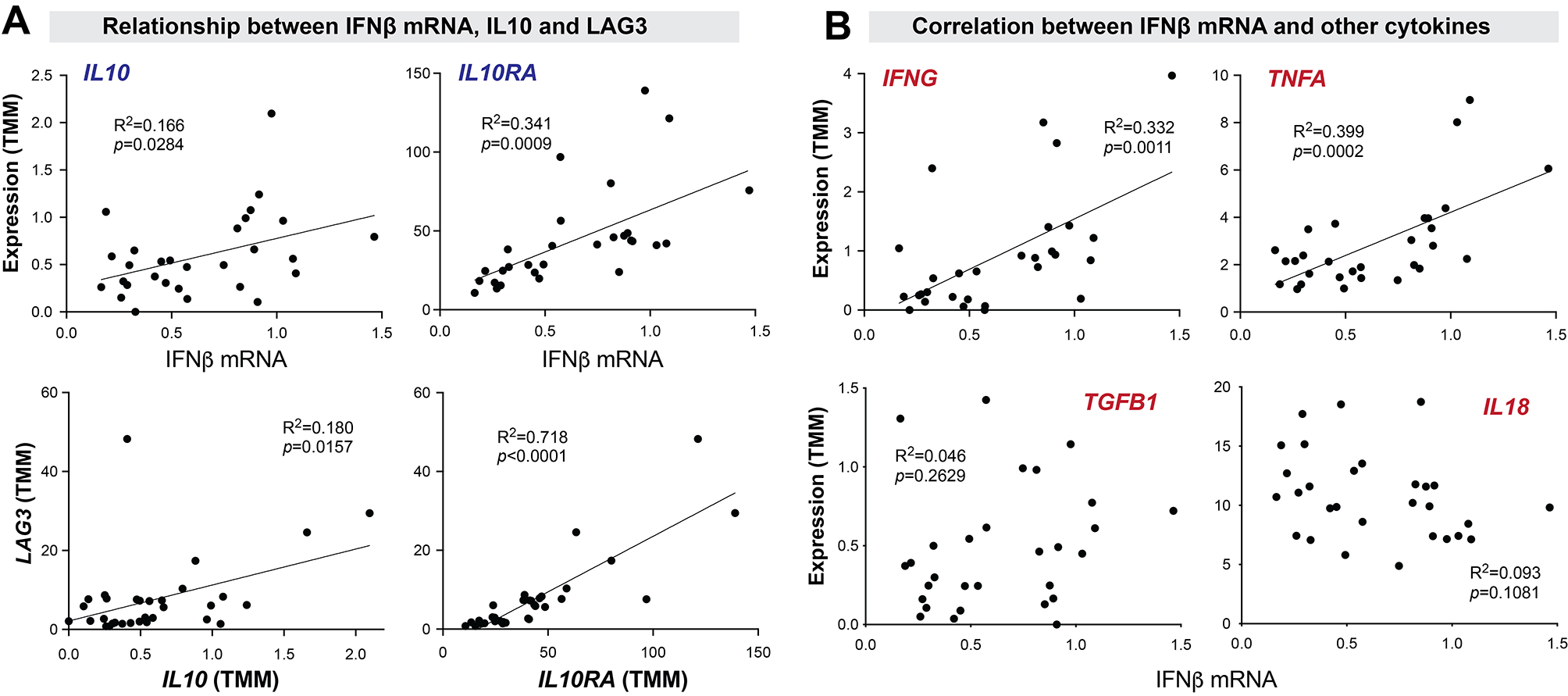

Supplement: S7 Fig — Linear regression plots are shown for (A) IFNβ mRNA levels, IL10, IL10RA and LAG3; and (B) IFNβ mRNA levels and cytokines IFNG, TNFA, TGFB1 and IL10. IFNβ mRNA levels were obtained previously [38]; the remaining genes were TMM values from the RNAseq experiment described here. R2 and p-values are shown for these relationships. (JPG) [file ppat.1008986.s007.jpg]
