# Supplementary material for: Qualitative Differences Between the IFNα subtypes and IFNβ Influence Chronic Mucosal HIV-1 Pathogenesis
Source: PLoS Pathog. 2020 Oct 16;16(10):e1008986. doi: 10.1371/journal.ppat.1008986 (PMC7592919; doi:10.1371/journal.ppat.1008986)
Supplement: S5 Table — (PDF) [file ppat.1008986.s012.pdf]

Table S5. IFNα subtype-specific IRGs are unlikely to be regulated by other IFNα subtypes.

	**Unique genes for each IFNα subtype**				
	**IFNα1 (N=110)**	**IFNα2 (N=36)**	**IFNα5 (N=201)**	**IFNα8 (N=48)**	**IFNα14 (N=257)**
**Total genes**					
**IFNα1**					
P-value		0.269 [0.000690, 1.00]	0.208 [1.08e-06, 1.00]	0.145 [0.000847, 0.905]	0.266 [9.28e-05, 1.00]
FDR		0.934 [0.0398, 1.00]	0.884 [0.000119, 1.00]	0.830 [0.0465, 1.00]	0.930 [0.00716, 1.00]
**IFNα2**					
P-value	0.199 [4.25e-06, 1.00]		0.137 [3.09e-05, 1.00]	0.191 [0.00391, 0.867]	0.116 [0.000231, 0.983]
FDR	0.951 [0.000467, 1.00]		0.906 [0.00280, 1.00]	0.947 [0.158, 1.00]	0.873 [0.0156, 1.00]
**IFNα5**					
P-value	0.150 [5.37e-07, 1.00]	0.274 [0.000482, 1.00]		0.271 [0.00120, 1.00]	0.526 [0.000603, 1.00]
FDR	0.747 [5.57e-05, 1.00]	0.871 [0.0240, 1.00]		0.868 [0.0474, 1.00]	0.967 [0.0281, 1.00]
**IFNα8**					
P-value	0.173 [4.14e-07, 1.00]	0.232 [0.000550, 0.935]	0.266 [0.000662, 1.00]		0.300 [0.00317, 1.00]
FDR	1.00 [6.61e-05, 1.00]	1.00 [0.0471, 1.00]	1.00 [0.0540, 1.00]		1.00 [0.200, 1.00]
**IFNα14**					
P-value	0.239 [4.01e-05, 0.979]	0.299 [0.00254, 0.971]	0.397 [0.000398, 1.00]	0.283 [0.0234, 0.891]	
FDR	1.00 [0.00489, 1.00]	1.00 [0.171, 1.00]	1.00 [0.0370, 1.00]	1.00 [0.613, 1.00]	

*Rows are the median and [min,max] p-value or FDR for the genes in that column, but in the other IFNα subtypes
