# Supplementary material for: Qualitative Differences Between the IFNα subtypes and IFNβ Influence Chronic Mucosal HIV-1 Pathogenesis
Source: PLoS Pathog. 2020 Oct 16;16(10):e1008986. doi: 10.1371/journal.ppat.1008986 (PMC7592919; doi:10.1371/journal.ppat.1008986)
Supplement: Attachment — Submitted filename: Guo PLOS Pathogens Rebuttal.docx [file ppat.1008986.s021.docx]

**Reviewer #1:**

Overall, this study provides extensive new information on the gene-regulatory activities of different IFN-I’s as they relate to HIV-1 infections. A main and well substantiated conclusion of the study is that after normalizing concentrations of IFN-I’s for IFNAR signaling strength, there remained substantial differences in the interferomes obtained from primary gut CD4 T cells, indicating qualitative differences among the different IFN-I species. The immunomodulatory activities predicted from the type-specific interferomes also varied among the different IFN-I’s. The inversion of the beta-interferome during chronic HIV-1 infection of the gut is an interesting observation. The study is carefully performed and presented and there are only relatively minor criticisms of the work.

**We thank Reviewer 1 for the encouragement and positive feedback.**

IFNbeta affinity for IFNAR2 vs ISRE activity should be included (or referenced) in the paper. Are the greater numbers of IRGs with IFN-beta related to a greater affinity for IFNAR2? Since there is speculation on the pathogenicity of IFNbeta in chronic HIV-1 infections this point needs to be further addressed in the paper.

**We thank Reviewer #1 for these constructive comments. The Lavoie et al *Cytokine* 2011 paper did not include IFNβ but its binding affinity relative to other IFNαs could be gleaned from previous reports (new references 42 and 43). Using the IFNAR2 KD value of 0.1 nM for IFNβ (ref. 43), the correlation between IFN-I ISRE-activity and binding affinity was lost (new Fig. 2E; lines 202-206). This further emphasizes potential qualitative differences between the IFN-Is (Discussion, lines 515-517).**

**As suggested, we tested if IFNAR2 binding affinity correlated with the number of IRGs and ISGs. The high binding affinity of IFNβ drove a strong correlation between binding affinity and the number of genes altered or induced (R^2^>0.94, p<0.01; lines 248-249). However, within the IFNα subtypes only, there was no correlation. It is indeed possible that the strong binding affinity of IFNβ drove the expression of additional genes relative to the IFNα subtypes despite not inducing higher ISRE activity (Discussion, lines 534-536). Evaluating mutants with binding affinities that are intermediate between the IFNα subtypes and IFNβ may be necessary to further address this potential impact on the number of regulated genes.**

**Reviewer #2:**

In the manuscript by Guo et al., they investigate the differential transcriptomes and anti-HIV activities induced by differing Type I IFN subtypes focusing primarily on IFNA2, IFNA14 and IFNB. The primary methodology used in this study is RNA-seq, which is employed to show qualitative differences in the interferomes induced by IFNβ and 5 different IFNα subtypes. The questions addressed by the authors – understanding the differential activities of the varying Type I IFNs is important for basic immunology– despite many years of study, there is still no consensus as to why the mammalian system utilizes so many IFNA genes, and if they have differing functional consequences. The scientific question has translational importance as several clinical trials are underway to test the efficacy of IFNA as a therapeutic to lower the HIV reservoir – and studies that clarify the biological mechanism of action and probe the differences between IFNA subtypes are needed.
The authors performed a series of in vitro HIV inhibition studies testing the relative activity of IFNAs and IFNΒ and demonstrate that IFNΒ and IFNA14 have higher anti-HIV inhibition activity compared to IFNA2. In addition, they performed RNA-Seq on lamina propria mononuclear cells testing the gene expression signatures of several IFNAs and IFNΒ in which they define “core IFNA interferomes” and “β-interferome”. They also performed RNA-seq of ex vivo gut CD4+ cells from individuals with untreated chronic HIV infection showed the presence of a strong type I IFN response, where there was an upregulation of 90% of genes overlapping with that observed in the “core IFNα interferome”. In contrast, the “β-interferome” is “inverted” with most associated genes downregulated during chronic HIV-1 infection. The authors demonstrate that IFNβ mRNA in the gut of chronic HIV is positively correlated with immune activation/inflammation and exhaustion while negatively correlated with genes that protect against pathogenesis. They show that the tested IFNα subtypes induce a core set of 266 genes while IFNβ specifically induces a broader interferome. The data also shows an incomplete overlap between the interferomes, suggesting qualitative differences between the subtypes.
The paper had several strengths: the use of primary lamina propria mononuclear cells (rather than PBMCs) for in vitro testing is commendable; additionally, the use of 19 HIV+ infected patients to 13 uninfected patients for ex vivo RNA-Seq comparison is reasonably powered. The synthesis and testing of varying IFNA subtypes is commendable.

**We thank Reviewer 2 for the positive feedback.**

The study suffered from several important weaknesses, discussed in detail below: (i) in general, several of the conclusions were not supported by the data (see details in Major Issues) (ii) the primary results of the study, reporting and comparison of differing “interferomes” by differing IFNA, was based on the in vitro experiments, which were significantly underpowered. (iii) On their own, the data from the transcriptome studies is underpowered and does not provide strong support for the conclusions, however, the authors are further attempting to use the data to clear up prior work by them and others that is somewhat controversial (i.e. do differing IFNAs have distinct biological activities or simply differing binding affinities for the IFNAR)? – this places an additional burden of reducibility that is not met by the underpowered nature of the study.

**In order to determine a suitable sample size for a study, a reasonable expected outcome is required. Our expected outcome is that canonical ISGs – particularly *ISG15*, the ISRE of which is the basis of the iLite assay – should be induced by all six IFN-Is tested. Our strategy captured not only *ISG15*, but >250 canonical ISGs as induced by the six IFN-Is (Fig. 4B, Table S3). If our sample size was insufficient, then the majority of these known antiviral ISGs should not have been detected.**

**That said, we agree that any study could always use additional donors, but unfortunately this was complicated by the nature of the primary cells we work with. Compared to the use of PBMCs which are readily accessible, gut LPMCs are more challenging to obtain. To increase reproducibility, we minimized cell type heterogeneity by utilizing *purified* CD4 T cells. We also noted that it’s possible that our data cut-offs may not be stringent. Thus, data with different fold-change cut-offs (2.0x, 2.5x, 3.0x) were presented (Fig. S3). In all scenarios, we found a substantial number of ‘outlier genes’ specific to an IFNα subtype. Additional evidence noted below further support that the distinct interferomes we identified were relevant.**

1. Throughout the manuscript, the authors make the argument that IFN anti-HIV activities are due to differing biological activities (i.e. gene expression), not simply IFNAR binding affinity. They show the level of residual virus replication at maximal dose of IFN-β to be 38.4% which they describe as “potent” – however, they also show that IFNA2, which the authors have published extensively on as “non-potent”, has a vRES of 39.6%. The IC50 is ~10X stronger for IFNΒ – which they use as a basis for its selection as “potent”, however this would argue that potency driven by affinity for IFNAR, not by differences in antiviral gene expression programs. Additionally, these experiments have broad variation in the data, and no statistics are given even though the experiment is relatively well powered (6 donors).

**We do not dispute that quantitative differences in binding affinity can drive the differences in antiviral activity *in vitro*. In fact, this was an important point in our previous studies (Harper et al, PLOS Pathogens 2015; Lavender et al J Virol 2016; lines 512-513). However, *in vivo,* there are clearly other factors involved as distinct immune responses can be induced by distinct IFNα subtypes (Lavender et al J Virol 2016). Our main goal here is to determine if there are qualitative differences that may influence how these IFNs should be deployed in the clinic. One way to address this is through unbiased transcriptomics.**

**The IFNα2 and IFNα14 data were already published (Lavender et al, J Virol 2016), and in that report, we used *average values* of the 6 donors at each dose to calculate the IC50 (line 144). Here we extended these data to include IFNα8, IFNα1 and IFNβ. We agree that another way to calculate IC50s and Vres is to determine it for individual donors. A limitation of this strategy is that for a few donors, mainly for IFNα1 (n=2), IFNα2 (n=1) and IFNβ (n=1), the IC50 was not reached. We used the highest IFN concentration as the IC50 for these donors. The data are now included as Fig. S1, Lines 771-781. IFNα2 was still less potent than IFNα8 and IFNα14. Vres were not significantly different from each other using data from individual donors, but the average Vres for each IFN-I tested matched what was presented in Figure 1 (Results, lines 160-164). We removed any discussion on the Vres as a potential indicator of qualitative differences between the IFN-Is.**

2. In the section “Identification of Novel IFN-Regulated Genes (IRGs) based on RNAseq Profiling”, the main claim is again not substantiated by their data (figure 4). This experiment was very low powered (n = 3) and used a low FC cut-off (1.5) to identify genes associated with each of the interferon subtypes.

**Please see response above.**

The relatively high proportion of genes identified that did not overlap with the INTERFEROME Database (30%) is cause for concern.

**As can be seen in Table S2, most of these ‘novel’ genes were noncoding RNAs or genes with tentative designations. This may be the reason why these were not included in the INTERFEROME database as of yet (lines 251-252).**

However, even more worryingly, the authors perform simple overlap analysis of DEGs from each IFNA subtype to define IFNA-specific “signatures.” Simple venn/overlap analyses can very easily misleading, particularly for underpowered studies – RNA-Seq benchmarking studies have shown that even for well-powered (n = 15) replicate experiments using the same set of input RNA, overlaps of DEGs defined by fold-changes and FDRs – the concordance of gene lists are in the range from 50-75%.

**We couldn’t find a specific reference for 15 replicate experiments required for RNAseq studies, so we were unclear how to respond. As mentioned earlier, we validated our study based on the detection of canonical ISGs, particularly ISG15.**

Here, the lack of gene list overlap is being interpreted as distinct biological activities. This is a central point, as Figures 5-7 are based on this concept. Several conclusions are drawn: (i) the identification of novel IFN-regulated genes was highlighted as one of the most important findings in this section of the manuscript, (ii) the authors provide these data as further evidence to resolve the “debate” about IFN-I activity being due to biological activity vs. IFNAR affinity. These conclusions cannot be reliably drawn from the data.

**We took an empirical approach and examined the ‘unique’ gene list for each IFNα subtype. These were defined as not significantly differentially expressed in any of the other IFNα subtypes. The reviewer was concerned that these genes may be in these unique lists due to chance and the low sample size in this study. To explore the possibility that these unique genes may still be showing differences in the other IFNα subtypes, we took the unique gene list for each subtype and calculated summary statistics on the FDR values for the other subtypes (for 5 IFNα subtypes x 4 other IFNα subtypes = 20 comparisons – see new Table S5, Results lines 277-282). If there was signal in these gene lists in other IFNα subtypes, then we may expect to see smaller FDRs. However, in almost all cases the median FDR was at least 75% (and usually above 90%), which was substantially larger than the FDR cutoff of 20%. This provides support that the genes that were only differently expressed in a specific IFNα subtype, were unlikely to show differences in the other IFNα subtypes. Furthermore:**

1. **IFNα14 altered 243 unique genes. Ingenuity Pathway analysis suggested that IFNα14 activated immune pathways – data from our previous study in humanized mice (Lavender et al, J Virol 2016) showed that IFNα14 could activate NK cells but not IFNα2.**
2. **IFNα5 significantly altered 201 unique genes (Fig. 4B) and weakly downregulated >360 genes that IFNβ and other IFNα subtypes upregulated (Fig. 4C, Table S7).**
3. **IFNβ induced more genes than IFNα – a finding that was previously reported, though we noted that the IFNs were not normalized against an ISRE reporter but with Units/ml.**

**We added a statement in the Discussion (lines 531-539) about these critical findings.**

3. The manuscript relies too heavily on correlation analyses, which are relatively weak arguments for the claims put forward by the authors.

**In contrast to mouse studies where genes can be knocked out, or nonhuman primate studies where compounds can be immediately inoculated, in the absence of clinical trials, human studies rely initially on correlations to formulate workable hypotheses that can be later explored through more direct interventions. Our studies reveal potential pathways associated with IFNβ that could be evaluated if IFNβ-blockade strategies will be tested in clinical trials. We believe these concepts are worth reporting as it could be relevant to other infections – for example, the data could have important implications for HBV and COVID-19 (Discussion, 616-624), where IFNβ (as well as IFNα2) is currently being investigated as a therapeutic agent.**

In addition, the final two figures, showing a correlation between IFN-β and markers of inflammation, microbial translocation and gut CD4+ T cells is not a novel finding.

**We previously published that IFNβ mRNA levels in the gut were associated with various clinical parameters (Dillon, Guo et al AIDS 2018). In contrast, Figures 8 and 9 (right panels) refer to the association of IFNβ-specific genes with IFNβ, inflammation, MT and gut CD4 T cells. These are novel analyses. No other studies to our knowledge has sought to evaluate IFNβ-specific genes in chronic HIV infection in the gut.**

In addition, too much emphasis on the unfamiliar concepts: “core-interferome”, “β-interferome”, “inversion of the β-interferome” and weakness of the associated experiments diminishes the interest in these findings.

**These are unfamiliar concepts because these are the novel findings of the work. To make the study more understandable, we had to coin terms to refer to ISGs that were induced by the 6 IFN-Is we tested (core) and ISGs that were induced by IFNβ (beta-interferome) but not the other IFNα subtypes we tested. Using terms such as ‘ISGs that were induced by the 6 IFN-Is we tested’ and ‘ISGs that were induced by IFNβ but not the other IFNα subtypes tested’ would have been too wordy to place in figure panels and would be challenging for the reader. We removed the term beta-interferome and replaced with ‘IFNβ-specific ISGs’ (lines 340, 378, 392, 403).**

4. There is a lack of unsupervised analysis such as PCA where clustering of genes/pathways associated with each of the individual interferon subtypes would have provided stronger data to show significant differences between the interferomes. The focus on analysis of individual genes carry less meaning than extensive scrutiny of the data, such as gene set enrichment analysis, which is more supportive of the biological relevance of their observations.

**The current study is focused on the gut, which has elevated IFNβ but downregulated IFNα subtypes (Dillon et al AIDS 2018). Thus, we focused our analyses on IFNβ-specific genes. Ingenuity Pathway Analysis indeed highlight potential pathways unique to IFNβ (Fig. 5B). Although a single PC1 value for IFNβ-specific genes can be used in correlations, we opted for individual gene analyses to capture the percentage that correlated with clinical parameters and importantly, gain potential mechanistic insights. By contrast, in the blood, only certain IFNα subtypes were induced. We are performing RNAseq on blood samples from the clinical cohort, and using PC1 values specific to each IFNα subtype for precisely this purpose. In other words, the current study is simplified by the fact that IFNβ is the major IFN-I in the gut.**

5. A major thrust of the introduction and discussion seemed aimed at resolving the debate with their prior study, some examples of the literature cited in the introduction was not an adequate representation of the current understanding of the interaction between HIV and interferon.

**We included additional references probing the role of Interferons in HIV-1 latency (references 6-11 and 33), Introduction lines 75-78, 108-110.**

The context for this study was given as a very narrow focus, with the emphasis on the authors’ past research.

**We disagree that this work has a very narrow focus. It addresses issues in the field re: whether IFN subtypes trigger distinct gene signatures and how distinct IFN-Is may be detrimental during chronic HIV-1 infection. These data have implications for how we view the diverse type I IFNs that can be further investigated using other cell types (blood CD4 T cells, dendritic cells, etc) and other viral infections. In fact, such studies are now ongoing with this work as the underlying basis.**

**Reviewer #3:**

Discussion stresses points that could have been addressed in data analysis:
(a) Downregulation of IFN-signaling by USP-18, UNC93B1. Was the expression of these genes explored relative to core or IFN-b specific gene expression as an explanation of the inversion shown in Fig 8? Is USP-18 or UNC93B1 strongly inversely correlated to IFN-beta expression as part of summary data shown in Fig 8A?

**We are very grateful to Reviewer 3 for this important suggestion. *USP18* and *UNC93B1* are core ISGs that are involved in negative feedback regulation of the IFN-I response (Table S3). In chronic HIV-1 infection in the gut, IFNβ mRNA levels positively correlated with *UNC93B1* but not *USP18*. *USP18* levels did not correlate with any of the IFNβ-specific ISGs. By contrast, *UNC93B1* levels inversely correlated with majority (88%) of IFNβ-specific ISGs in the gut. We now include new Figures 7D and 7E and Results, lines 394-401 and 410-412 showing these associations, and suggest that *UNC93B1* induction by IFNβ may be a potential mechanism for downregulation of IFNβ-specific ISGs in the gut (Discussion, lines 607-609).**

(b) Link between microbial translocation, IFNb, and ISGs - was there any indication of activated myeloid gene expression?

**We previously evaluated CD40 MFI in CD1c+ mDCs in the colon biopsies to evaluate myeloid DC activation (line 420; Dillon et al, Mucosal Immunology 2016). Unfortunately, we found no significant correlations with IFNβ and ISGs and have included this in the revised Table S11. It is possible that other activation markers would correlate that can be explored in future studies (Discussion, line 584-586).**

Authors describe a relationship between IFN-b expression and LPS, but not sCD14? Was sCD163 tested?

**We agree that it is odd that IFNβ associated with LPS but not sCD14. Unfortunately, data on sCD163 is not available at this time but this is an important point that we could probe in future studies (Discussion, line 584-586).**

(c) As done in previous report by same group, discussion raises the link between blocking IFN-b during the LCMV infection and the potential for IFN-b to be pathogenic in HIV infection - yet IL-10 expression (raised as mechanism in LCMV induced by IFN-I signaling) or its relation to LAG3 in inhibiting T-cell responses is not directly addressed data shown.

**We thank the reviewer for this comment. IFNβ mRNA levels indeed positively correlated with *IL10* (R^2^=0.13, *p*=0.02) and *IL10RA* (R^2^=0.34, *p*=0.0009) expression in the gut. Furthermore, the levels of *IL10* and *IL10RA* expression also positively correlated with *LAG3* levels. These important points are now included in the Results (lines 466-469), new Figure S6 (panel A) and Discussion (lines 592-594).**

1. Please define LPMC (line 130) as did not see it define before.

**Addressed as requested (line 133).**

2. Please add reference to "previous study" in legend line 183

**Addressed as requested (line 189, ref. 12)**

**Reviewer #4:**

Type I interferons (IFN-I) are key cytokines that contribute to control viral infections. They can block HIV-1 replication by inducing or repressing the expression of a cascade of interferon-regulated genes (IRGs) and stimulating innate and adaptive immune cells. IFN-α can reduce viral RNA in HIV-1–infected humans and SIV–infected animals, but may also exert deleterious effects during HIV-1 infection, especially during later stages of infection.
Distinct IFN-I have different effects on HIV-1 replication and this paper by Guo et al. performed transcriptome analysis on: 1) CD4+ T cells purified from the gut of healthy controls (n=3) and stimulated ex vivo with individual IFN-Is; 2) colon biopsies from HIV+ individuals and HIV uninfected controls. They showed that genes induced by IFNβ in gut CD4 T cells ex vivo were downregulated in the gut during chronic HIV-1 infection. They conclude that their findings reveal qualitative differences in interferome induction by diverse IFN-Is and suggest potential mechanisms for how IFNβ may drive HIV-1 pathogenesis in the gut.

This work presents unique data of the gut response to IFN-I and manages to specifically assess the qualitative effects of the different IFN-I in their induction of IRGs measured by bulk RNA sequencing. Nevertheless, there were a number of concerns with this study. The following are a few important scientific arguments to take in consideration to support the conclusions of the manuscript.

**We thank Reviewer 4 for the constructive feedback.**

1) It is quite unclear why the authors switched from working with LPMCs and CD4 T cells in the different assays and render the results quite difficult to interpret.

**We routinely use LPMCs for antiviral inhibition assays as they’re more straightforward to work with. However, for RNAseq analysis, the cell type heterogeneity of LPMCs (on average, 65% CD4 T cells, and the remaining are CD8 T cells, DCs, B cells, ref. 44) could lead to confounders. Thus, we opted to select the most abundant cell type in LPMCs and the cell type where HIV-1 and antiviral ISGs are likely to interact. These points were now noted in the Results, line 214-217.**

2) In Figure 1 the HIV-1 inhibition curve of IFN-b is obtained using LPMC. Do the authors find the same results with isolated CD4 T cells and does combination of different IFN-I even lower the level of residual HIV-1 replication? Other cells in LPMCs will respond to IFN-I and may also have an effect on viral replication.

**We thank Reviewer 4 for this important point. In previous work, depletion of CD11c+ and CD11c/CD19+ from LPMCs did not alter HIV-1 replication (as measured by p24 ELISA) (Dillon et al, J Immunol 2012). However, depletion of CD11c+ cells in LPMCs abrogated bacteria-mediated enhancement of HIV-1 infection. It is certainly possible that other cell types could affect how IFN-Is could inhibit HIV-1. For example, IFN-Is could upregulate CD169/Siglec1, which is a mediator of HIV-1 trans-infection. However, the main purpose of the transcriptomics study is to evaluate if there were differences in the ‘interferomes’ between the various IFNα subtypes and IFNβ. Using total LPMCs would have introduced cell type heterogeneity as a confounder (see our response above).**

In addition, the time of IFN-I addition is inconsistent between the results and the material and methods part. Was the IFN-I added 1 day after infection or at the same time as infection?

**For the HIV-1 inhibition assays, the IFNs were added at the same time as the infection as we previously reported (Harper et al PLOS Pathog 2015). For RNAseq, we evaluated gene expression 18 h post-treatment. A full cycle of HIV-1 replication is thought to occur within 24-48 h (new references 45 and 46), so evaluating ISG induction at 18 h should capture relevant antiviral mechanisms during the viral replication cycle (Results, lines 222-224).**

3) The interpretation of some of the paper is affected by a lack of description of the method used to quantify the % of CD4+ T cells among CD45+ cells in colon biopsies? Did the authors purify the cells? This does not appear to be the case.

**We apologize for this omission. In the previously published clinical study (Dillon et al, Mucosal Immunol 2016; Dillon et al, Mucosal Immunol 2014), up to 30 colon pinch biopsies were obtained from each of the participants. About 20 pinch biopsies were pooled for same-day flow cytometric immunophenotyping (hence the %CD4+ cells among CD45+, as well as CD40 MFI in CD1c+ mDCs) and the rest were frozen for histology (FFPE and OCT) and transcriptomics (RNAlater). This is now clarified (Results, lines 414-418).**

4) The authors mentioned the level of different IFN-I in the gut, but other pro-inflammatory cytokines are also expressed such as IL-6 or TNF-a. What is the level of these cytokines (transcript or protein level) in their colon biopsies? Do these cytokines induce a similar set of gene expression?

**We thank the reviewer for this suggestion. We mined the transcriptomics dataset for cytokines. Just like the IFNα subtypes and IFNβ, the RNAseq had insufficient depth for many cytokines including IL6. However, sufficient signals were obtained for *IL10, TNFA TGFB1, IL18* and *IFNG*. IFNβ mRNA levels (measured previously via qPCR; Dillon et al AIDS 2018) significantly correlated with transcript levels of *IL10, TNFA* and *IFNG* obtained via RNAseq. These points are now included in the new Figure S6 and Results, lines 466-470 and Discussion, lines 588-594. We currently do not know how much overlap in gene signatures IL10, TNFα and IFNγ would elicit in primary gut cells; this would be an interesting subject for a follow-up study.**

5) In Supplemental Figure 1, LAT genes is not consistent between the RNAseq and the qPCR results. The authors should normalize the data to IFN-a14 to better assess whether the different IFN-I induces the same level of IRGs.

**We normalized based on mock treatment as that provides a better indication of fold-induction by the IFN-Is. The *LAT* data on IFNα14 in Figure S2 may be a technical error, as one sample had a 7.013-fold increase relative to mock, whereas the 2 others had fold changes of -1.000 and -0.440. We apologize to the Reviewer as it appears that the large error bar was accidentally deleted during initial figure preparation. Unfortunately we do not have additional samples for reanalysis by qPCR, and so we just modified the figure to highlight the data point that drove the positive fold-change in this particular sample, as well as noting the discrepancy in the caption (lines 793-795).**

6) Figure 4B is quite confusing. The authors described 1257 IRGs but only 903 genes are shown in the Euler diagram.

**The 903 genes correspond to just the core IRGs and the unique IRGs for each IFN. IRGs that were shared between 2 to 4 IFNs were not included. We added a note in the Figure 4 caption to clarify this point (lines 303-306).**

7) In the heatmap of Figure 4C, the genes described as differentially regulated by IFN-a5 are not all supported by the Supplemental Table S4. The authors should clarify this point.

**We thank Reviewer 4 for this insightful point. The genes in Table S4 were based on our IRG criteria of at least 1.5 fold-difference relative to mock and a 20% FDR cut-off. The 367 genes that appeared to be downregulated by IFNα5 but were induced by IFNα1, 2, 8, 14 and IFNβ were based on the average values and were gleaned from the heatmap in Figure 4C. Although the genes that were differentially downregulated by IFNα5 were not statistically significant based on our IRG criteria (hence not appearing in Table S4), the large number of genes differentially downregulated by IFNα5 was striking and suggested that IFNα5 may be qualitatively different than the other IFN-Is. These were now clarified in Results, lines 285-288. We also included a new Table S6 (line 834) listing down these genes as they could be useful for follow-up investigations on unique aspects of IFNα5 biology, as also predicted by Ingenuity Pathway analyses (line 322, Figure 4D).**
