# Supplementary material for: Qualitative Differences Between the IFNα subtypes and IFNβ Influence Chronic Mucosal HIV-1 Pathogenesis
Source: PLoS Pathog. 2020 Oct 16;16(10):e1008986. doi: 10.1371/journal.ppat.1008986 (PMC7592919; doi:10.1371/journal.ppat.1008986)
Supplement: Attachment — Submitted filename: Guo PLOS Path Rebuttal 2.docx [file ppat.1008986.s022.docx]

**Reviewer #2:** My original concern with this study is that the primary finding: that IFN subtypes induce diverse functional responses – is primarily based on severely underpowered RNA-Seq experiments (n =3) where the lack of overlap of DEGs is the “proof” that the responses are truly divergent.

    A number of studies have now estimated the sensivity (i.e. “power” of RNA-Seq to detect “true” DEGs at sample sizes.

    I have listed several of these studies and their findings below.

    1. Wu et al: PROPER: Comprehensive Power Evaluation for Differential Expression Using RNA-seq

    Bioinformatics 2015 Jan 15;31(2):233-41

    These findings showed that using RNA-Seq n = 3, the overall power to detect truly DEGs was 0.27 (human samples) and 0.62 (inbred mice). Even more worrying was that the false-discovery cost at n =3 was 0.94 (meaning for every DEG found, a false-positive was found) and 0.32, respectively.

    2. Schrurch et al: How Many Biological Replicates Are Needed in an RNA-seq Experiment and Which Differential Expression Tool Should You Use?

    RNA.053959.115v1

    This study estimated for edgeR based analyses that at n = 3 the power to detect significantly expressed genes was 25% (Figure 1A). and they provide guidelines:

    “At least six replicates per condition for all experiments.”

    “At least 12 replicates per condition for experiments where identifying the majority of all DE genes is important.”

    3. Hart et al: Calculating Sample Size Estimates for RNA Sequencing Data

    Journal of Computational Biology v20, #12, 2013

    Figure 3 – in the best datasets with low biological CV n7 = 7 for highly expressed genes, for datasets with higher CV, n = 40.

    4. Yu et al. Power analysis for RNA-Seq differential expression studies.

    BMC Bioinformatics 2017 18:234

    Figure 6: at n = 5 the power to detect DE’s was < 20%.

    5. Ching et al. Power analysis and sample size estimation for RNA-Seq differential expression.

    RNA 20: 1 – 13.

    Six datasets were simulated for power estimates. At n = 3, three datasets had power less than 50%. Three had power ~80% for n = 3 – but these were data in which two diverse tissues were compared (UHR vs Brain reference RNA, cancer cells vs normal tissues, stem cells vs fetal head) and the median log fold change of DE’s was extremely large fold-change: 2.13, 3.33, 2.1 with very small CV.

    Cumulatively – these studies have demonstrated that if the goal of an RNA-Seq study is to detect all or most of the truly differential expressed genes – then sample sizes need to be larger, usually above n = 10. With an n = 3, the power to detect the DE’s is usually around 30%. Additionally, it comes with a high rate of reporting false positives, which would be more likely by the increased FDR of 0.2 reported in this study.

    My main criticism of this study is that a significant conclusion (IFNs induce different programs of gene expression) is based on underpowered RNA-Seq experiments where the lack of overlap is to be expected. The most logical explanation for the lack of overlap is a simple Type II statistical error.

    I had previously pointed this out as major drawback, and this issue hasn’t been addressed.

**We thank Reviewer #2 for the references and again highlighting this important point regarding sample sizes. False negatives due to low power can indeed result in the lack of overlap, thereby affecting our conclusions. Unfortunately, it was not possible to add samples to increase power since it is difficult to find gut donors with large enough numbers of cells to purify primary gut CD4 T cells for the 7 independent treatments required (mock, 5 IFNα subtypes and IFNβ). As an alternative way to address this issue, we added S5 Table in the last revision to show that the genes that were not overlapping were not even close to our significance cutoffs (relatively large p-values/FDR) for the other IFNα subtypes. For example: each of the 201 genes that we noted as ‘IFNα5-specific’ was considered significant at a 20% FDR cut-off for IFNα5. When these genes were evaluated against the gene induction datasets for IFNα1, 2, 8 and 14, majority had FDR values >90%. In other words, these genes were not even close to being statistically significant for the other IFNα subtypes. The same analyses were performed for IFNα1-, IFNα2-, IFNα8-, and IFNα14-specific genes. The median FDR values against the other IFNs for these specific genes were mostly >90%. This gave us confidence that we are less likely to make false negative statements. To more clearly illustrate this point, we included a new S3 Figure, lines 279-285 to supplement S5 Table on how unlikely it was for the IFNα5 and IFNα14-specific genes to be significantly induced by the other IFNα subtypes.**

**The power analyses cited by the Reviewer are generally useful, but their value for our study are unclear as these were from unrelated studies. Statistical power that is not defined in the context of effect sizes or fold changes is difficult to interpret, more so to calibrate other studies. For example, the reviewer’s statement that “With an n = 3, the power to detect the DE’s is usually around 30%” is not meaningful unless attached to a specific differential expression level or fold change. We previously noted that we observed these IFNα-subtype specific genes with a variety of fold-induction cut-offs (from 1.5 to 3.0-fold; S4 Figure) further strengthening the notion that our detection of IFNα-subtype specific genes were unlikely due to random chance.**

**Another critical factor in power calculations is the variability of the data. Often, studies that promote general guidelines use mixed cell types (such as *in vivo* patient samples), but our samples are more homogeneous (purified CD4 T cells) and treated in a controlled fashion *in vitro* with IFNs. This approach should reduce variability in the data, as compared to, say, using bulk mixed lymphocytes or analyzing patient specimens. In fact, over 240 genes, including the gene promoter we used to calibrate the IFN doses, *ISG15*, were stimulated by all 6 IFNs tested (core ISGs), providing an internal positive control for our approach. These are now noted in Discussion, lines 535-542.**

**Finally, 70% of the IFN-regulated genes we identified have already been defined in the literature and were deposited in the INTERFEROME database (Fig 3D). The 30% that were not in the INTERFEROME database were genes that were noncoding or had tentative designations at this time (lines 250-252). Overall, these gave us confidence that we are capturing authentic genes regulated or not regulated by IFNs.**

    I suppose an alternative would be to take the current data and conduct a power analysis to demonstrate if reasonable sensitivity could be expected in their dataset. Several R packages such as PROPER exist that are used often on publicly available datasets and could easily be implemented here.

**Due to inherent bias, post-hoc power analyses are not recommended in the statistical community. Please see guidelines noted in the references below:**

**https://gpsych.bmj.com/content/32/4/e100069**

**https://accpjournals.onlinelibrary.wiley.com/doi/abs/10.1592/phco.21.5.405.34503**

**https://journals.lww.com/annalsofsurgery/Citation/2019/01000/Don_t_Calculate_Post_hoc_Power_Using_Observed.46.aspx**

**Hoenig and Heisey (2001) The Abuse of Power: The Pervasive Fallacy of Power Calculations for Data Analysis**

**However, we do note that the data accumulated from this work could be used to estimate sample sizes for future studies of other cell types – for example, peripheral blood samples where donors and cell numbers are not going to be a logistical issue. In fact, these follow-up studies are currently in progress (Discussion, lines 569-573).**

    The data are currently in the SRA but the annotated dataset should be deposited to NCBI GEO for compliance with open source genomics data sharing.

**We deposited the data in NCBI GEO as suggested. Accession numbers (likely a SuperSeries number for all the datasets in this study) have not yet been generated but we needed to resubmit before the deadline. These accession numbers will be made available prior to publication (lines 774-775).**

**Reviewer #3:** Revised paper has improved clarity but a few minor clarifications are still needed:

    1. As understanding IFN-b relative to other IFNs is main point of report, unclear why IFN-b data is omitted from Fig 4 Panels C and D.

**We focused Figure 4 on just a comparison of the IFNα subtypes. In Figure 5, the comparison between the IFNα subtypes and IFNβ was highlighted.**

    2. IFN-beta data also missing from Table S3 lacks so harder to interpret relative to Table S7 where it is included.

**We now included a column in Table S3 to showcase the fold-induction values for IFNβ.**

    3. What is the difference between IFN-beta RNA summarized in Fig 7D and that in Column D of Table S12? USP-18 is shown as not correlated in Fig7D but positively correlated in row 88 of S12 with core ISGs in similar fashion as UNC93B1 in row 32?

**We thank Reviewer 3 for catching this error. The previous Figure 7D utilized a simple linear regression, while the data in S12 Table is more robust as it accounted for co-variates (e.g., age and gender). We removed Figure 7D-E and revised lines 401-402 to refer to S12 Table that both *USP18* and *UNC93B1* correlated with IFNβ mRNA.**

    4. As paper centers on the potential role of USP-18 and UNC93B1, it would be useful to add supplementary Tables listing genes that are correlated with these two negative regulators as proposed contributors to the IFN-beta-specific inversion in tissue. As USP-18 is associated with direct negative regulation of IFNR2 signaling whereas UNC93B1 is tied to TLR functionality and signaling, listing the correlation and overlap between these two genes and core versus IFN-beta-specific genes may aid future investigation of what may be distinct negative regulatory pathways.

**We added a new S13 Table showing the PWH-altered Core ISGs (n=126) and IFNβ-specific ISGs (n=112) that correlated with *USP18* and *UNC93B1*. To account for multiple testing within the four sets of correlations, we calculated the FDRs from the p-values of t-tests for each correlation coefficient, and we report the Pearson's correlation coefficients for relationships that were significant at a 5% FDR threshold. Notably, none of the IFNβ-specific ISGs correlated with *USP18*. By contrast, 89% of IFNβ-specific ISGs negatively correlated with *UNC93B1*. These analyses suggested that *UNC93B1* may be contributing to the downregulation of IFNβ-specific ISGs in gut biopsies from chronic PWH (Results, line 402-404, S13 Table).**

    5. Discussion should mention (a) type III IFNs can also access IFNRs yet not addressed here as a potential source for sustained core ISGs, and (b) USP-18 has recently been linked to T-cell dysfunction.

**In the study preceding this (Dillon et al AIDS 2019), we were unable to detect type III IFNs in the chronic gut samples by quantitative PCR. This was noted in the Introduction, lines 122-124.**

    6. Added COVID-19 discusion text is speculative as not clear that there is IFN-beta compartmentalization in gut as in HIV infection and several reports of human trials have shown potential beneficial effects in spite of references listed by authors in munrine models. Surely a worthy area of investigation but not directly tied to points raised in this ms.

**We agree that it’s speculative. We note that the *Lancet* study showing a potential benefit of IFNβ limited their treatments to no more than 7 days post-onset of symptoms. It remains unclear if further delaying treatment with IFNβ in patients, or administration during severe COVID19, will be beneficial, but JAK inhibitors, which would inhibit not only IL6 but also IFNβ, are being tested against severe COVID19. We tempered the statement to suggest that studies of distinct IFNs in viral infections could have important therapeutic implications (Discussion, lines 635-640).**

**Reviewer #4:** 1) The core IRGs and beta-interferome described here are specific to gastro intestinal CD4 T cells of healthy donors.

    In line 362 the authors state “We emphasize that all these genes were induced by IFNβ in gut CD4 T cells ex vivo (S6 Table). Given that a great majority of these IFNβ-specific ISGs were downregulated in PWH, we conclude that a significant inversion of the ‘beta-interferome’ was observed during chronic HIV-1 infection in the gut”.

    However, during HIV-1 infection, depletion and changes in the frequency of the different CD4 T cell subsets in the gut occurs. How consistent are the RNAseq assay with CD4 T cells from gut of HIV-1 infected patients?

**We thank Reviewer 4 for this comment. Chronic HIV-1 infection leads to a significant decrease in CD4+ T cells in the gut and could certainly be a confounder in our analyses, as noted in the Discussion lines 581-582. However, we observed that Core ISGs were upregulated in the gut samples from chronic PWH – we would expect these genes to have also been downregulated if it was due to an artifact of lower numbers of CD4 T cells in PWH. These points were now included in Discussion, lines 582-586.**

What are the frequencies of the different CD4 T cell subsets in the gut (Th17, Th22, Treg, etc…)? Do they have the same level of IFNAR? Do they respond the same way to the different IFN-I at the authors’ optimal concentration?

**The impact of IFNs on distinct Th cell subsets – particularly from the gut – has not yet been evaluated to the very best of our knowledge. This would be a very important angle to pursue in future studies. We recently reported that IFNAR2 expression was not altered in mucosal immune cells (including T cells, NK cells and B cells) in PWH (Ickler et al, PLOS One 2020), as we now note in lines 586-589, reference 81.**

Assessing the expression of these genes in CD4 T cells of HIV-1 infected patients would provide more relevant information.

**Unfortunately, we do not have viable cells left from the gut from this cohort. As we mentioned in our earlier response, the initial flow cytometry results already required pooling ~20 pinch biopsies. At that time, technologies such as CyTOF are not yet routine in many laboratories. That is why we focused our efforts in characterizing the transcriptome from bulk tissue. The current study should inform the design of such studies that will focus on individual cell types.**

 Also, do the authors observed difference when comparing the transcriptome of CD4+ T cells not stimulated with IFN-Is and the transcriptome of colon biopsies of HIV-negative controls?

**This would be an interesting comparison to make in a future clinical study. Unfortunately, the donors we used as sources of CD4 T cells in the in vitro study were from different from that of the clinical study. Based on PCA analysis (S1A Figure), the transcriptome of primary CD4 T cells from the gut is highly donor-dependent, so it would be important to perform this interesting experiment suggested using samples from the same donors.**

    2) Related to the previous point, it is rather difficult to interpret the RNAseq data between the in vitro study and the ex vivo study. The in vitro study is done on total CD4 T cells from the gastrointestinal tract of healthy controls and the ex vivo study is done on colon pinch biopsies directly. The reference 44 shows that the frequency of CD4 T cells is 65% of viable CD45 cells in a set of LPMCs. However, the frequency of colon CD4 T cells provided in Table 1 is inconsistent with this finding. It ranges from 5.21 to 23.35% of CD45 in HIV-1 infected patients and from 20.44 to 49.89% of CD45 in healthy donors. Please provide an explanation for this discrepancy.

**The differences in frequencies likely relate to the different protocols used in processing the clinical pinch biopsies versus the discarded surgical tissue samples. For the pinch biopsies, tissue samples were collected and immediately digested in collagenase media to prepared single cell suspensions for subsequent flow cytometry analysis. Therefore, the total cell suspension will contain all cells in the pinch biopsy including any mononuclear cells in the epithelial layer. By contrast, isolation of LPMC from surgical samples is performed using a multi-step protocol including removal of the epithelial layer and associated cells prior to collagenase-digestion of the underlying tissue. Thus, differences in frequencies based on percentages of viable, CD45+ cells will occur due to the differences in the fraction of total CD45+ cells.**

This broad range of variation in the % of CD4+ T cells may explain the inconsistent ISGs induction with IFN-I observed between the in vitro and the ex vivo studies. Moreover, the frequency of CD4 T cells being lower than 50% of CD45 cells in all biopsies, therefore the results from the in vitro RNAseq cannot be used as it is to infer that IFN-b may drive HIV-1 pathogenesis.

**We agree that this is a confounder of our analysis as we noted in our response above. However, we already had previous data that IFNβ is upregulated in the gut in PWH (Dillon, Guo et al, AIDS 2019), and our current study further extends this finding towards IFNβ-specific genes.**

1) In Figure 1 the HIV-1 inhibition curve of IFN-b is obtained using LPMC. Do the authors find

    the same results with isolated CD4 T cells and does combination of different IFN-I even lower

    the level of residual HIV-1 replication?

**We have not performed an experiment at this time looking at IFN sensitivity in isolated gut CD4+ T cells. Indeed, it would be interesting to observe how combinations of IFNs influence replication. We performed such an experiment with IFNα14 (a potent antiviral IFN) and IFNα1, which appears to bind to IFNAR2 but not IFNAR1. However, our mixed- IFNα1/IFNα14 experiments did not provide evidence that IFNα1 serves as a competitive antagonist of IFNAR signaling. However, from the current work, we observed some interesting properties of IFNα5, which appears to downregulate genes that were upregulated by other IFNα subtypes. There are important follow-up questions that we are pursuing related to IFNα5 due to this work (Discussion, line 535).**

    2) Please change the line 142 if the IFN-Is were actually added at the same time as the infection.

**We thank Reviewer 4 for catching this error. This was corrected.**

    3)Please change the line 438-439, “blood CD4 T cell counts were more significantly associated with IFNβ-specific ISGs (87% negatively correlated) than core ISGs”. Actually, they are positively correlated. The author should discuss this finding as type I IFN is known to mediate CD4 T cell depletion (Sivaraman V et al. J Virol 2011).

**Corrected as noted and the new reference was included in the Discussion, lines 631-633.**
